# CiteSpace-based global science, technology, engineering, and mathematics education knowledge mapping analysis

**DOI:** 10.3389/fpsyg.2022.1094959

**Published:** 2023-01-10

**Authors:** Duo Yang, Xiaopeng Wu, Jiali Liu, Jincheng Zhou

**Affiliations:** ^1^School of Computer and Information, Qiannan Normal University for Nationalities, Duyun, China; ^2^Faculty of Education, Northeast Normal University, Changchun, China; ^3^School of Mathematics and Statistics, Qiannan Normal University for Nationalities, Duyun, China; ^4^Key Laboratory of Complex Systems and Intelligent Optimization of Guizhou Province, Duyun, China

**Keywords:** STEM education, bibliometrics, knowledge mapping, CiteSpace, knowledge graph

## Abstract

To better understand the latest developments in global science, technology, engineering, and mathematics (STEM) education research, this study collected STEM education research materials to sort out the development of STEM education as a whole, so as to get a clearer path and trend of STEM education development. This study conducted a visualization and quantitative analysis of the literature on STEM education research in Science Citation Index Extended (SCI-E) and Social Science Citation Index (SSCI) using the CiteSpace (5.8.R3) tool. First, the basic information of STEM education was analyzed in terms of annual publication volume, authors, countries, and research institutions. Secondly, the main fields, basic contents and research hotspots of this research were analyzed by keyword co-occurrence and keyword time zone mapping. Finally, the research frontiers and development trends are presented through co-citation clustering and high-frequency keyword bursts. The research hotspots are focused on engineering education, teachers’ professional development, and gender differences. The research frontiers are mainly related to teacher professional development, 21st century skills, early childhood creativity, and gender differences. This study systematically analyzes the latest developments in global STEM education research, which is beneficial for readers to understand the full picture of STEM education research so that researchers can conduct more in-depth studies and promote better development of STEM education. The number of analyzed literature is limited. We only analyzed articles from SSCI and SCI-E databases, and the articles were written in English. In addition, we only analyzed the literature and lacked empirical studies on the findings of the literature.

## 1. Introduction

The impact of the fourth industrial revolution has led to significant changes in the way people learn. Traditional single-field education and targeted education oriented to productive work can no longer meet social needs. The current education system is based on department instruction, which is not suitable for developing students’ creativity and ability to solve practical problems. Science, technology, engineering, and mathematics (STEM) education originated in the United States (US) and is short for the initials of four disciplines: Science, Technology, Engineering, and Mathematics (Marginson et al., 2013), focusing on the cross-fertilization of multiple disciplines, but not simply superimposing the four disciplines, but forming an organic whole. In the 21st century, where information technology is developing rapidly, people are increasingly concerned about the development of science and technology, and the STEM education concept is known as the theoretical basis and practical means of technological innovation. STEM education is an organic whole driven by the task of solving practical problems, enabling students to apply and acquire knowledge in practice and develop problem-solving skills and innovative thinking (Barrett et al., 2014). STEM education, as a comprehensive and innovative talent cultivation model, has received widespread attention from the international education community, has been widely implemented in the global education field, and has also achieved remarkable results. STEM education is also related to future talent development and national competitiveness. In response to the challenge of the lack of innovative talent, the United States has pioneered STEM education. Since STEM education became a national development strategy in the United States, there has been a wave of reform in STEM education around the world. STEM education has become an important way for countries to develop science and technology innovation and talent training. As the country where STEM education originated, the National Science Board (NSB) adopted the *Undergraduate Science Mathematics and Engineering Education*, also known as the *Neal panel’s report*, in 1986 (Shapley, 2000). This is the first policy document on STEM education in the US and became the beginning of the development of STEM education. Since then, in the development of STEM education, the United States government has enacted a series of laws and policies to ensure the implementation of STEM education. STEM education has also gained great attention in China as a new educational paradigm for a new era. At the beginning of the 21st century, theoretical research on STEM education began in China. Since 2015, STEM education in China has entered a phase of rapid development. China has not only proposed relevant policies to elevate STEM education to the level of national strategy, but also made rapid progress in terms of practice and theoretical research (Min et al., 2020). This is also the case in other developed countries such as the United Kingdom and Australia, which have made the development of STEM education and the cultivation of innovative talents a strategic national goal (Irwanto et al., 2022). At present, national researchers in STEM education are still exploring the development path of the concept integration of STEM education, further interpreting the connotation of STEM education, and pointing out the direction for the global cultivation of high-level talents in the new era (Bo, 2022).

In terms of education policies, countries around the world provide strong guarantees for the development of STEM education. The United States pioneered STEM education in the *Neal panel’s report* issued in 1986 in order to cultivate innovative talents and improve the country’s competitiveness. Since the introduction of STEM education in the United States, STEM education has become the focus of education reform in various countries as a change in the way of education. As a leader in STEM education, the United States has enacted a series of policies on STEM education, providing institutional guidance and financial security for the development of STEM education. For example, to promote the development of STEM education, the United States Senate enacted the *America Creating Opportunities to Meaningfully Promote Excellence in Technology*, *Education*, *and Science Act* (also known as the *America COMPETES Act*) on August 2, 2007, which clearly stipulates STEM teacher training, financial security, and other issues, and has become the leading document to promote the rapid development of STEM education (Stine, 2009). After more than 30 years of practical exploration, to achieve high-quality and more equitable development of STEM education, the National Science and Technology Council released *Charting a Course for Success: America’s Strategy for STEM Education* in December 2018, known as the “STEM-Education-Strategic-Plan” (Li and Schoenfeld, 2019). The plan lays out a vision for the development of STEM education in the United States over the next 5 years and a path to achieve it. Finland is an innovative country, and the LUMA (LUMA is the Finnish word for STEM) program was introduced in the 1990s with the goal of “STEM for all” to strengthen STEM education practices and enhance students’ interest in STEM education (Vihma and Aksela, 2014). The United Kingdom formally included STEM education in government documents in 2002, and in January 2017 the government issued the *Building Our Industrial Strategy: Green Paper*, which identified technical education as central to the development of modern industry in the United Kingdom, as well as the need to address the shortage of STEM skills (Parsons, 2002; Allwood and Skelton, 2017). The *Dresden Resolution* of 2008, entitled “Progress through Education - The German Qualification Plan,” confirmed the importance of MINT (MINT is the German acronym for Mathematics, Information Technology, Natural Sciences, Technology.) education and made it an important goal for educational development (Blättel-Mink, 2009). Australia enacted the *STEM in the National Interest Strategy* in 2013, which for the first time proposed the development of STEM education at a national level and set targets for the development of STEM education in Australia from 2013 to 2025 (Corbett, 2014). China released the *China STEM Education: White Paper* on June 20, 2017, proposing the “China STEM 2029 Plan” (Su, 2017). The White Paper summarizes the effectiveness and problems of STEM education implementation in China and sets out the goals to be achieved by 2029 for STEM education in China. The release of the white paper has pointed out the direction for the development of STEM education in China, and these documents have provided policy assurance for the development of STEM education.

With the advent of the information age and knowledge economy era, the ability to innovate in science and technology has increasingly become a key element of the core competitiveness of countries around the world. As an educational model for cultivating innovative talents, STEM education has received attention and importance worldwide, and more and more countries have invested in the reform of STEM education. The importance of STEM education is reflected in the fact that countries such as the United States, Australia, Germany, Finland, China, and United Kingdom have all introduced corresponding policies to support the development of STEM education. For example, on January 31, 2006, the United States government released the *America COMPETES Act*, which sets out the ambitious goal of leading the world with innovation, aiming to enhance the United States competitiveness and innovation capacity on a global scale by strengthening investment in the field of science and technology (Gonzalez, 2015). On October 30, 2007, when the Soviet Union was celebrating the 50th anniversary of the successful launch of the first artificial satellite, to reflect on the threat of 50 years ago, the National Science Board again issued the report *National Action Plan: Emergency Notice to Address the* United States *STEM Education System* to warn that the United States must enhance the creativity of its citizens to make STEM education grow smoothly (Augustine, 2007). In order to promote sustainable and stable economic development and to cope with the shortage of highly skilled personnel, the German federal government, together with the United States, has put forward initiatives to promote talent development in MINT education and to initiate MINT education programs at all levels of schooling and education through curriculum reform and new standards at the basic education level (Blättel-Mink, 2009). In November 2013, Finland established the LUMA National Center, which became an important symbol of the development of STEM education. The LUMA program is based on the principle of share and majors, it serves children and adolescents aged 3–19 years old by tailoring STEM learning and educational activities outside of school as a way to promote STEM education research and teacher development (Aksela, 2015). The Australian Federal and United States signed the *National Strategy for STEM Schooling (2016–2026)* with Territory Education Ministers at the Australian Education Commission meeting in December 2015, which aims to ensure that Australian students have the STEM-related knowledge and skills needed to succeed in a rapidly changing society of the future (Murphy et al., 2019). On June 7, 2016, China promoted the reform of educational modernization and informatization to advance comprehensive socio-economic development. The Ministry of Education has issued the *13th Five-Year Plan for Education Informatization*, which is another national policy document for the development of STEM education after the Ministry of Education’s *Draft for Comments*. The paper emphasizes interdisciplinary learning, especially to enhance students’ information literacy and innovation skills with the help of STEM education (Kennedy and Johnson, 2016). The United Kingdom believes that a major constraint on the country’s development at present is the lack of people with STEM skills and that the root cause is STEM education. Therefore, the United Kingdom has elevated STEM education to a strategic level for future national development (Smith, 2010). The above documents provide effective pathways and measures for the development of STEM education in a more systematic way.

STEM has not only attracted sufficient attention at theoretical and policy levels but also at practical level, where STEM education has been explored extensively. Developed countries such as the United States, Australia, Canada, and United Kingdom have emphasized the importance of having more people with high-level STEM skills (Aslam et al., 2018; Sharma and Yarlagadda, 2018; Irwanto et al., 2022), and each country has taken measures to support the development of STEM education. For example, STEM education is delivered by the United Kingdom public in the form of STEM activities in communities, museums, and informal science centers to encourage students to participate in STEM (Aslam et al., 2018). *The America Creating Opportunities to Meaningfully Promote Excellence in Technology*, *Education*, *and Science Act*, passed by the 110th Congress from January 2007 to January 2009, established several new programs. To enhance the teaching of the K-12, a Center of Excellence was established in each National Laboratory region. This center relies on the national labs and associated partner universities to promote summer institutes to improve teacher knowledge and proficiency. To foster interest in STEIM education, increase opportunities for young people to pursue STEIM careers, and give more students hands-on experience in STEM, the United States announced the start of “National Lab Week,” according to the United States White official website on February 28, 2016. During the first National Lab Week, more than 50 world-class federal laboratories in more than 20 cities were open to students. At the same time, the government was encouraging schools, libraries, and other community groups to organize similar events to provide more opportunities for students to experience STEM practices. According to a September 11, 2016 report on the *Washington Post* website, computer chipmaker *Qualcomm* has partnered with *Virginia Tech* to invest in the creation of Thinkabit lab. Located at the Northern Virginia Campus in Falls Church, the lab focuses on STEM classes. In addition, Qualcomm started the first Thinkabit Lab in San Diego a year and a half ago, serving 800 students from four other regions. Virginia Tech’s Thinkabit Lab was the first lab not located in California, and the lab was free and open to clubs (such as Girl Scouts) and all school districts in the Washington, DC area. The *Helmholtz Association of German Research Centers* is the largest scientific research group in Germany and has established 32 campus STEM labs for primary and secondary school students. Students can participate in campus labs in a variety of ways, from short theoretical sessions and hands-on experiments to ongoing research on their own or in their research teams over a longer period (from 1 week to 2 years). In China, many influential STEM education institutions and organizations have also emerged in society, such as S*hanghai STEM Cloud Center*, *China STEM Education Collaborative Alliance* (Su, 2017). Shanghai established the *STEM Cloud Center* in 2014 and then opened a *STEM Science and Technology Museum* in 2015 (Ruifang, 2017). In 2016, the *China STEM Education Collaborative Alliance* parsed STEM practice issues for participants from three dimensions: STEM curriculum design, STEM curriculum teaching implementation, and STEM teaching assessment (Ying, 2021). Jiangsu will pilot the first STEM education programs in 26 schools, and there will be more and more STEM programs on primary and secondary school campuses (Xiuling et al., 2017). In 2018, the *Shanxi STEM Education Collaborative Innovation Center* was established in Xi’an, and five schools in the province, including *Xi’an High-Tech International School*, were awarded STEM education pilot schools. East China Normal University, as an important base for teacher training in China, actively introduces the concept of STEM education and builds STEM labs. Taking the solution of water quality judgment engineering project as the guide and virtual simulation experiment as the carrier, students are guided to use the interdisciplinary knowledge of geography, physics, mathematics, and computer to develop intensive water quality optical comprehensive measurement virtual simulation experiment. The *China STEM Education Collaborative Alliance* advocates “A-STEM,” i.e., “humanities-led interdisciplinary education.” Since its establishment, the Alliance has held the annual *Science and Innovation Education Festival*, public service academic seminars and science teacher training, etc. It has also established experimental schools, model schools, and base schools throughout China.

STEM has been developed for more than 20 years, and has made great strides in both policy and theory, as well as in technology and practice, and has also accumulated a wealth of experience, providing an effective way to cultivate innovative and complex talents, while also providing a new breakthrough to the traditional education model. There is a wealth of material available on the study of STEM education, covering all aspects of STEM education development. Although there are some studies on the overall discourse and comprehensive review of STEM education, we cannot yet clearly see the path and trend of STEM education development in these studies. Therefore, by collecting rich materials of STEM research, this study tries to sort out the development of STEM education as a whole and tries to get a clearer path and trend of STEM education development. Therefore, this study will focus on the following three research questions:

1. What is the basic distribution of STEM education research, such as authors, countries, institutions, etc.?
2. What are the hot directions of STEM education research?
3. What are the trends in STEM education research?

## 2. Materials and methods

### 2.1. Data collection

Based on the WoS core collection platform, the SCI-E and SSCI databases were selected as data sources. The first article on STEM Education was recorded in April 2004. In order to minimize the omission of important basic research from earlier years, the data search was set to cover the period from April 2004 to April 17, 2022. A search on the topic of “STEM Education” or “STEAM Education” yielded a total of 1,420 relevant articles. This study extracts the year of publication, title, author’s country, author institution, abstract, keywords, citation frequency, and other information of each literature to analyze the research hotspots and development trends.

### 2.2. Analytical methods

#### 2.2.1. CiteSpace and setting

The data statistics and analysis tools used in this paper are mainly CiteSpace (5.8.R3) application software, which is a multivariate, time-sharing, and dynamic information visualization software developed and designed by Professor Chen Chaomei of Drexel University in the United States (Chen, 2006). The software is mainly used to study the research hotspots and frontiers in a certain field through high-frequency keywords, emergent terminology, and co-occurrence analysis of keywords in the literature, which can be used to analyze and predict the hotspots, evolutionary development history, and research frontiers and trends in the discipline. The selected 1,420 documents were imported into the CiteSpace application, with Time Slicing set to “April 2004–May 2022,” Years Per Slice set to 1 year, and the rest of the options set by default in the software.

The graph is mainly presented in the form of nodes and lines, with N denoting the number of network nodes and E denoting the number of network lines. The size of the nodes reflects the frequency of relevant data references or occurrences, the lines indicate the relationships between the nodes, and the thickness of the lines between the nodes reflects the strength of the connections between the data, which are not fully presented in the figure due to clarity and visualization (Wu et al., 2019). Aggregation effects are measured in terms of modularity and silhouettes. The value of Q represents the degree of modularity, and the value range of Q is generally [0,1], the larger the value, the better the clustering effect, if Q > 0.3, it indicates that the delineated clustering structure is significant. The network homogeneity evaluation index Silhouette (S), S ≥ 0.5 means that the result of clustering is reasonable, and as the value of S is closer to 1, it reflects the higher homogeneity of the network. Density indicates the network density (Liu et al., 2018).

#### 2.2.2. Paths of analysis

In order to comprehensively analyze the status and development trend of STEM education research, and based on the characteristics of CiteSpace, this study mainly analyzes its path from the following three aspects:

1. Basic information analysis of STEM education. This allows us to get an overview of STEM education in general, including the number of publications, authors, countries, institutions, etc.
2. Analysis of research hotspots in STEM education. The co-occurrence mapping of keywords and the time zone map are analyzed, which let us understand the main areas, basic contents, and research hotspots of STEM education research since 2004.
3. Analysis of research frontiers and trends in STEM education. A research frontier consists of a set of co-cited core papers and a group of current source papers that cite one or more of these core papers (Upham and Small, 2010). The co-cited references were clustered and analyzed, and the references that were cited more frequently were selected based on these clusters, and these articles were read closely to understand the current research frontiers in STEM education. Burst-detection algorithms are used to identify burst keywords regardless of the frequency with which their host articles are cited (Chen et al., 2012). Burstness refers to the intensity of the sudden appearance or disappearance of research subject keywords in a certain research field within a certain period, and some extent represents the direction of transformation of a certain research trend (Zhang et al., 2020). Based on the observed sudden changes of keywords in different periods, the trending research themes in different periods can be inferred.

## 3. Results analytical

### 3.1. Analysis of basic information on science, technology, engineering, and mathematics education

#### 3.1.1. Annual distribution of publications

**Figure 1:**
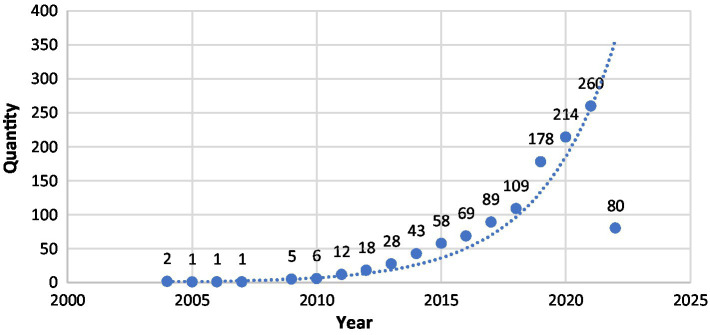
Annual distribution of the number of articles issued.

Counting the number of academic papers or academic journals in a field and analyzing them provides insight into the development of the field. The trends in the volume of literature show the changing trends in academic attention to research topics within a certain time frame (Zhang et al., 2020). According to the search results, 1,420 papers on STEM education were published from April 2004 to April 17, 2022, and the annual changes are shown in Figure 1.

According to the changes in the number of papers published, they can be divided into three stages of development, namely the budding stage, the slow development stage, and the rapid development stage. In the first stage (2004–2010), the budding stage, STEM education research was just starting, and the number of published papers were relatively small, averaging 2 papers per year. In the second phase (2011–2018), STEM education research developed slowly, with an incremental trend in the number of publications per year, and several research results emerged during this phase, with an average of 53 publications per year. The third phase (April 17, 2019–2022) is the rapid development phase, STEM education research is developing rapidly, the number of literatures increased by 69 in 2019, many research results are emerging in this phase, and the research results are more abundant. During the 3 years 2019–2021, the average number of publications per year is 217. Based on the fitting of Figure 1 for the period 2004–2022, it can be predicted that the number of articles will reach about 350 in 2022. This increasing trend year by year means that STEM education is attracting the attention of more and more scholars.

#### 3.1.2. Authors collaboration network map analysis

**Figure 2:**
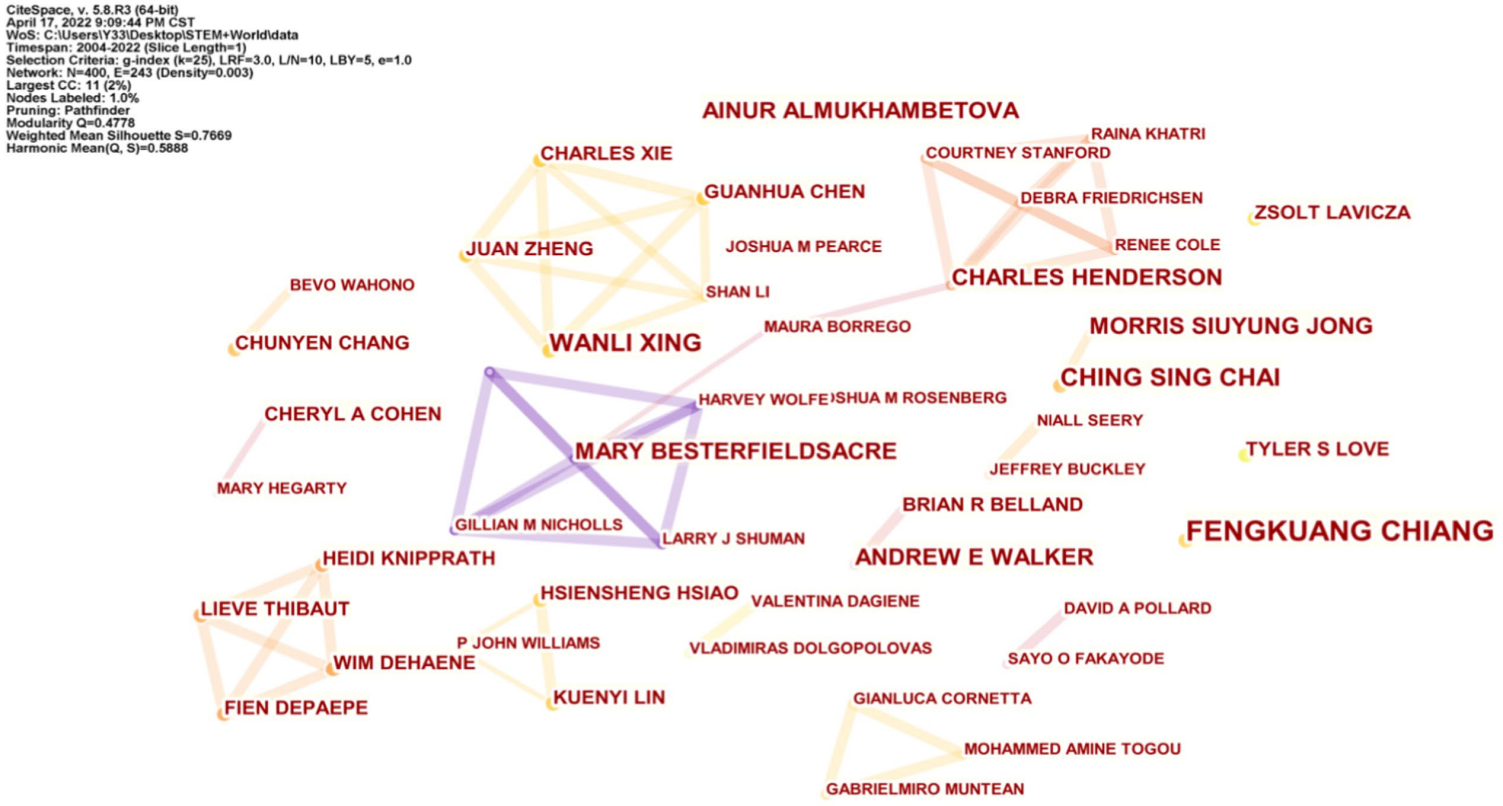
Authors’ collaboration network map.

The authors’ collaborative knowledge map reveals the main research forces in a given field (Gazni et al., 2012). Figure 2 shows the academic collaborations among authors engaged in STEM education research. As can be seen from the figure, there is a tendency for small author collaborations, with authors forming several academic research teams. There are 400 nodes and 243 lines connecting the nodes in the graph, indicating that between 2004 and April 17, 2022, there are more authors studying STEM education, but less collaboration between authors. Four research teams are working closely together on STEM education, among which two teams, CHING SING CHAI and WANLI XING, have published an equal number of articles and the most. The second-highest number of publications is by a research team with MARY BESTERFIELDSACRE as the core research team, and the third-ranked research team is centered on CHARLES HENDERSON. The field of STEM education research is characterized by low collaboration among authors (Density = 0.003) and few core authors in the field, with only eight authors have published more than two articles. Among them, FENGKUANG CHIANG is the most published person with six articles indexed in SCI-E and SSCI databases.

#### 3.1.3. The country of origin of the article

**Figure 3:**
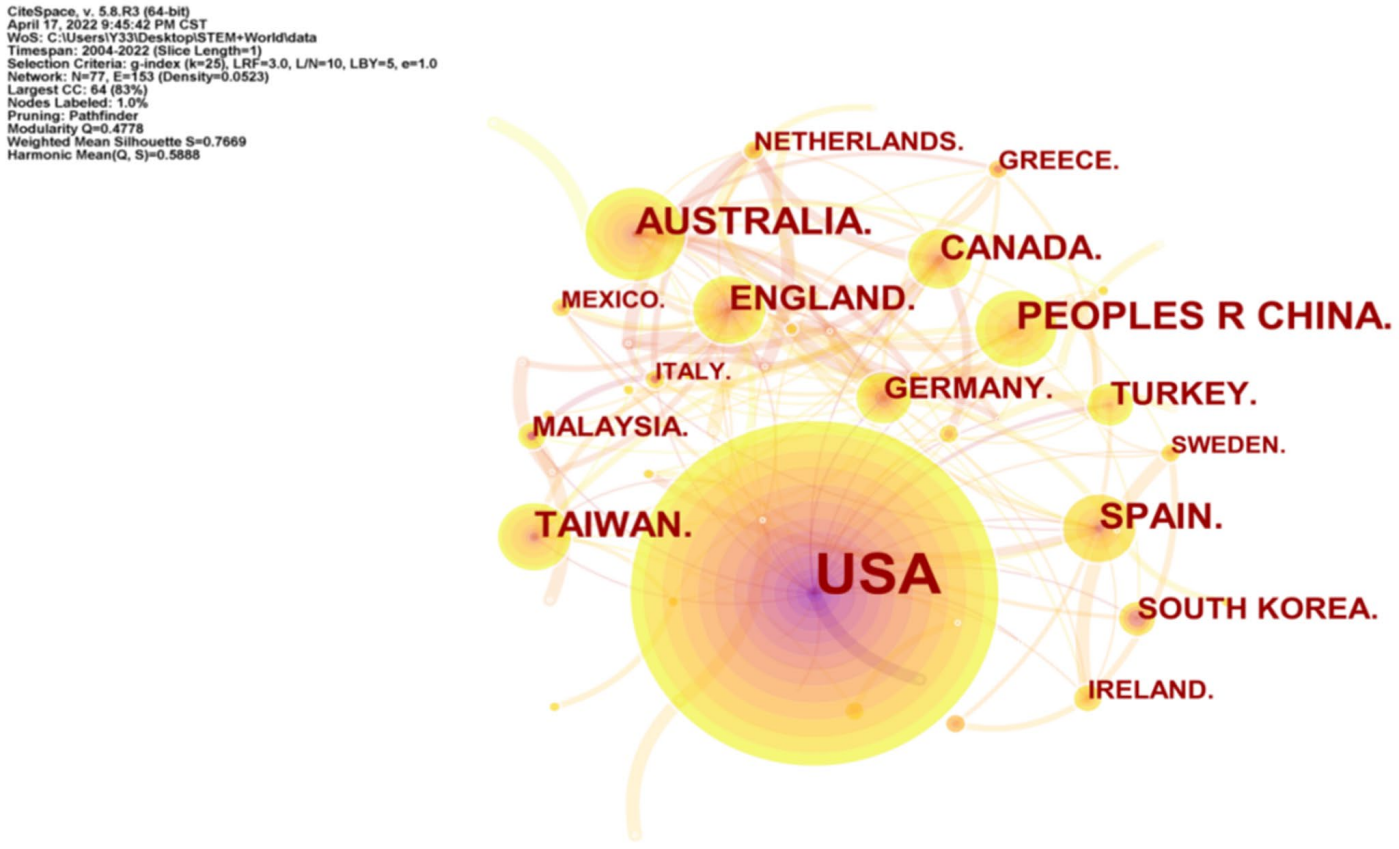
The country of origin of the article.

To understand the distribution of countries of origin of articles on STEM education, we obtained a network mapping based on author countries/regions through CiteSpace (Figure 3). The mapping shows 77 nodes and 153 lines connecting the nodes. A node represents a country/region, and the size of the node indicates the number of articles issued in that country/region. A line is created when two countries/regions have a cooperative relationship, and the thickness of the line reflects the closeness of the cooperation between the countries/regions (Tang et al., 2018). The number of nodes and lines in the graph reveals a relatively strong academic collaboration link between countries/regions of STEM education research (Density = 0.05). Table 1 shows the top 17 countries with the most published papers. In terms of the number of published papers, the main academic contributions come mainly from the United States, China, Australia, Taiwan, and Spain. In terms of the distribution of publications in the top 5 most productive countries, 632 came from the United States, followed by 70 from mainland China, 60 from Australia, 46 from Taiwan, China, and 45 from Spain. From the above data, it can be seen that American scholars have published the most articles, nine times more than second-ranked China. The United States alliances and organizations in STEM education research, as well as the formulation and promulgation of some policies, are of great significance to the research and development of STEM education and have been widely borrowed by other countries.

**Table 1:** Top 17 countries in terms of number of articles issued.

Number	Count	Year	Countries
1	632	2004	UNITED STATES
2	70	2014	CHINA
3	60	2011	AUSTRALIA
4	46	2013	TAIWAN
5	45	2015	SPAIN
6	41	2010	ENGLAND
7	38	2014	CANADA
8	33	2014	TURKEY
9	23	2011	GERMANY
10	22	2015	SOUTH KOREA
11	20	2014	MALAYSIA
12	15	2016	NETHERLANDS
13	13	2016	GREECE
14	13	2017	IRELAND
15	12	2015	MEXICO
16	11	2013	SWEDEN
17	10	2016	ITALY

#### 3.1.4. Research institutions

**Figure 4:**
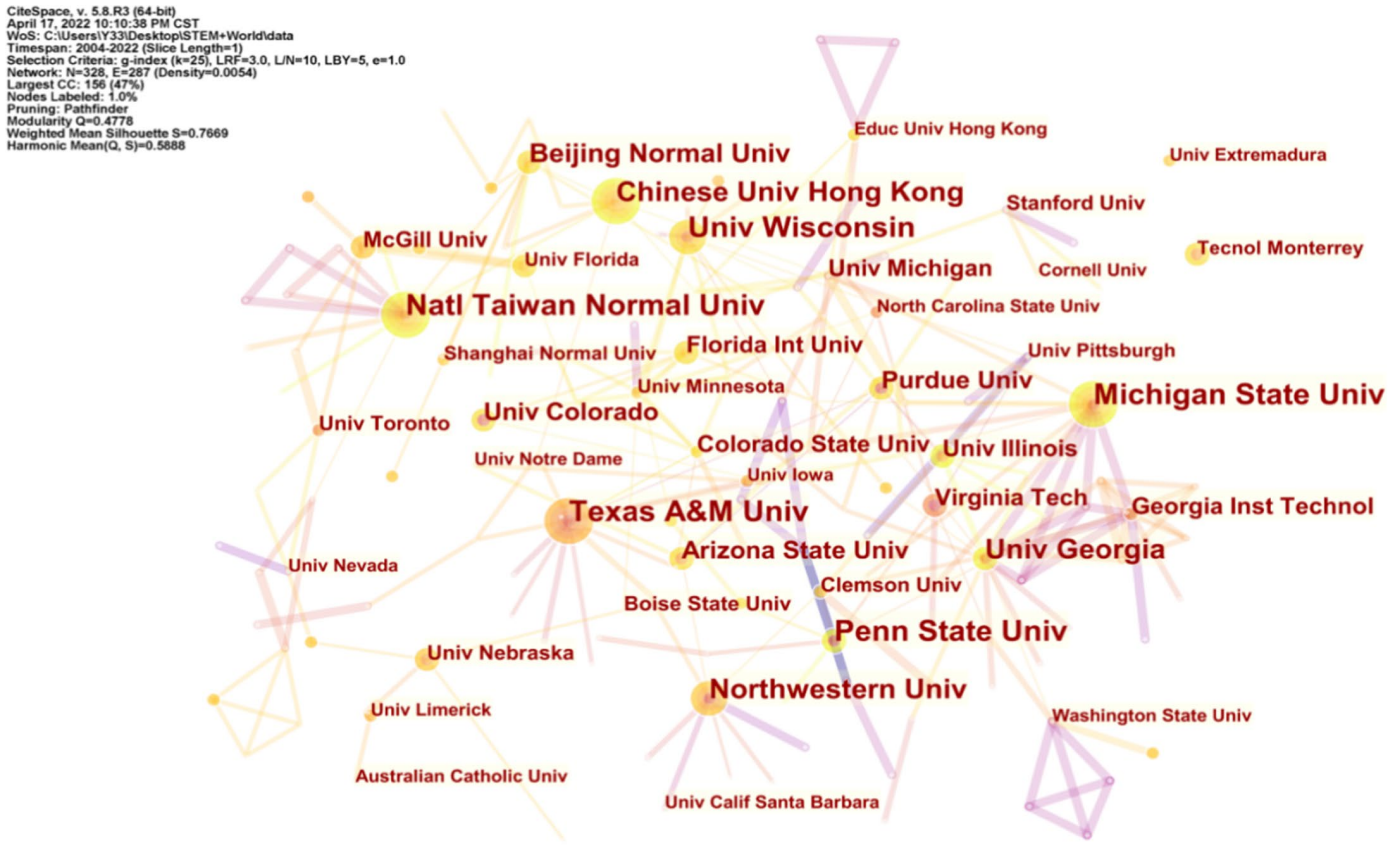
Major research institutions.

The number of papers published by a research institution reflects the research capability of the institution to a certain extent, and the statistical analysis of the number of papers published by research institutions can better reflect the development history and research results of each research institution (Danni et al., 2018). Figure 4 shows that there are 238 nodes and 287 lines of institutions conducting research, reflecting the academic collaborations between institutions engaged in STEM education research. Table 2 shows the research institutions with more than or equal to 10 publications. In terms of a number of publications, Texas A&M University, Michigan State University, National Taiwan Normal University, and University Wisconsin have made major academic contributions. Among them, Texas A&M University and Michigan State University published the highest number of papers, with 20 papers in SCI-E and Social SSCI databases. This was followed by National Taiwan Normal University with 19 publications and University Wisconsin with 18 publications. The importance and influence of the two research institutions, University Wisconsin and Chinese University Hong Kong, in the field can be reflected by their betweenness centrality. In terms of the top 16 research institutions, all of them are universities, signifying that higher education institutions are an important pillar of STEM education research.

**Table 2:** Research institutions with ≥10 publications.

Number	Count	Centrality	Year	Institution	Countries
1	20	0.09	2014	Texas A&M University	UNITED STATES
2	20	0.09	2012	Michigan State University	UNITED STATES
3	19	0.07	2013	National Taiwan Normal University	CHINA
4	18	0.1	2015	University Wisconsin	UNITED STATES
5	17	0.04	2004	Penn State University	UNITED STATES
6	16	0.05	2010	Northwestern University	CHINA
7	16	0.07	2012	University Georgia	UNITED STATES
8	15	0.14	2019	Chinese University Hong Kong	CHINA
9	13	0.06	2016	Beijing Normal University	CHINA
10	12	0.03	2012	Arizona State University	UNITED STATES
11	12	0.04	2011	Purdue University	UNITED STATES
12	12	0.01	2015	University Colorado	UNITED STATES
13	10	0.03	2006	Georgia Institute of Technology	UNITED STATES
14	10	0.02	2018	Florida International University	UNITED STATES
15	10	0.05	2012	Colorado State University	UNITED STATES
16	10	0.01	2015	University Illinois	UNITED STATES

Of the top 16 research institutions, 12 are in the United States and four are in China. In the United States, Texas A&M University and Michigan State University are tied for first place with the same number of publications. In China, National Taiwan Normal University is ranked first. A direct correlation between the number of papers published by researchers at research institutions and the number of papers in the countries where these institutions are located was derived from a comparative analysis (Wu et al., 2019). For example, researchers at Texas A&M University, Michigan State University, and University Wisconsin have published many articles on STEM education, which is one of the reasons for the high number of publications in the United States In addition, the scientific research ability of the United States is far superior to that of other countries because of the outstanding research capacity of the universities, the adequate funding, and state support for research, which are policies that are worthy of reference by all countries.

### 3.2. Analysis of research hotspots in science, technology, engineering, and mathematics education

**Figure 5:**
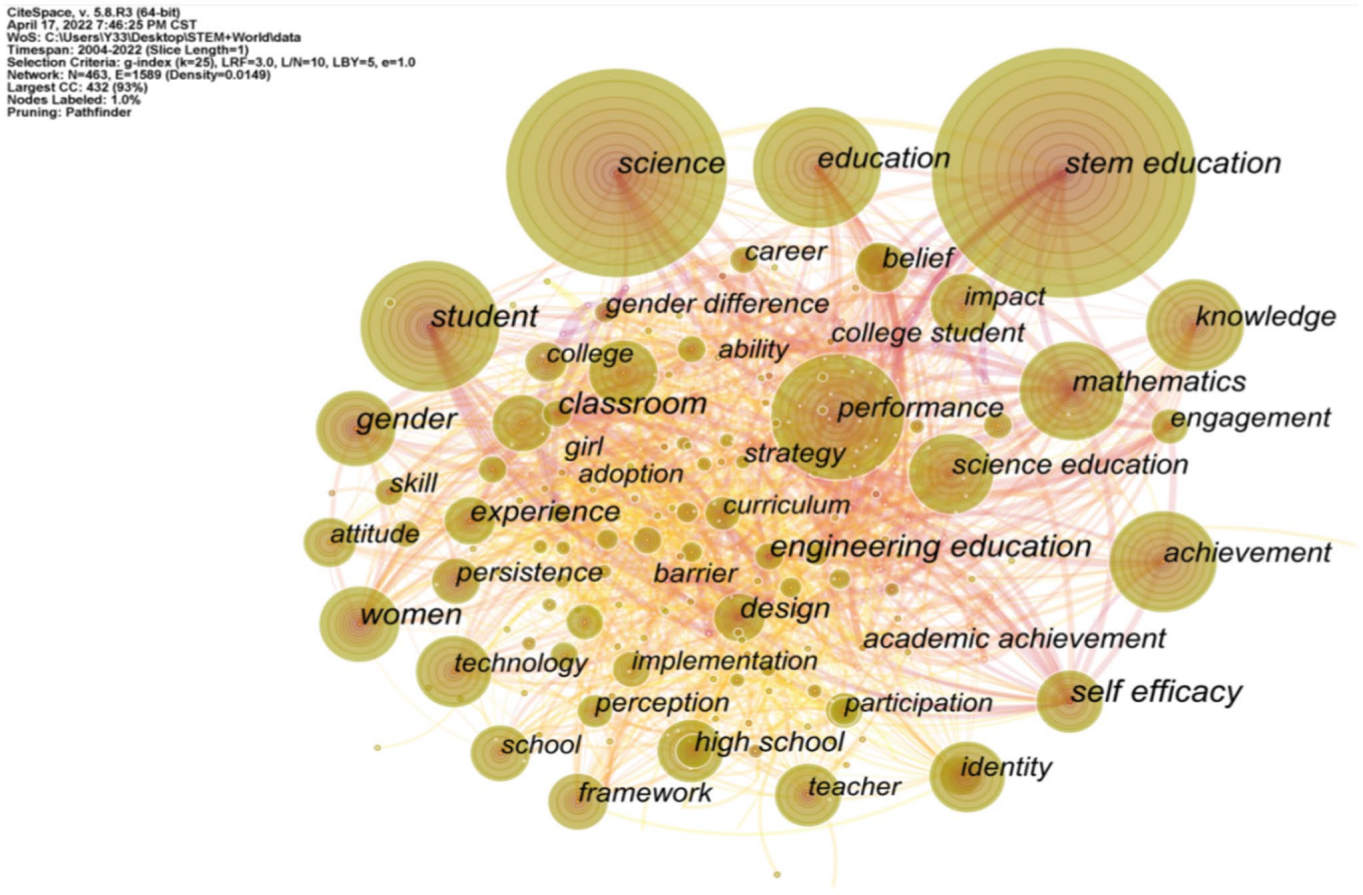
Keyword co-occurrence analysis.

**Figure 6:**
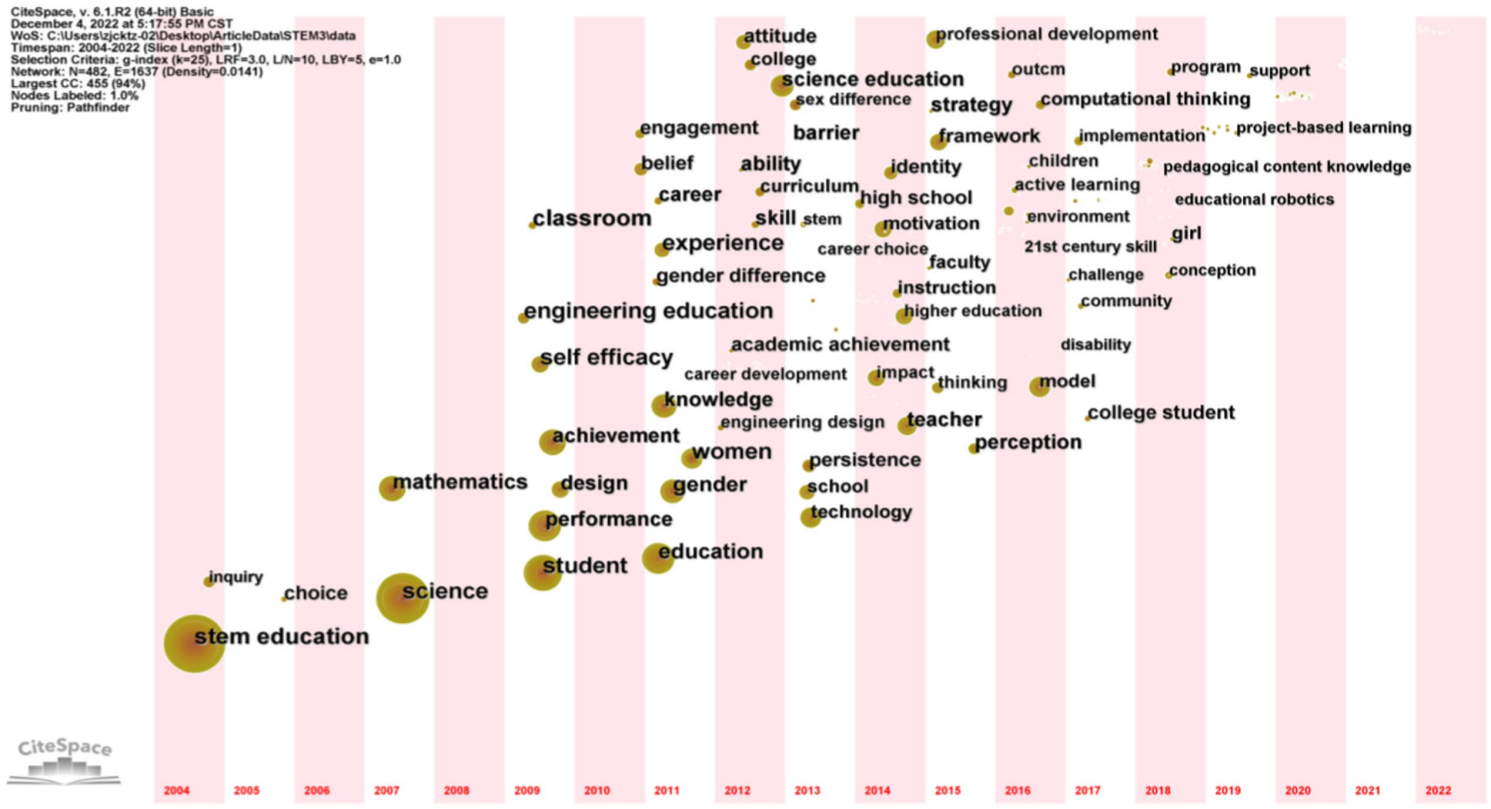
Keyword time zone graph (shows the keyword distribution from 2004 to April 17, 2022, with the time slice set to every year. Each circle in the graph represents a keyword that first appears in the analyzed dataset and is fixed in the first year. If the keyword appears in subsequent years, it will be superimposed on the first occurrence).

The keyword co-occurrence network reflects the hot and core research content of a field (Li et al., 2016). Figure 5 shows a keyword co-occurrence network with 463 nodes and 1,589 lines, where the nodes represent keywords. It can be seen that the font size of keywords is directly proportional to the co-occurrence frequency of keywords (Schneider, 2004). According to the results of keyword co-occurrence analysis, the four keywords with high frequency (frequency over 100) are “stem education,” “science,” “education,” and “student,” which appear 387, 273, 134, and 131 times, respectively (Table 3), so they can be considered as the main areas and basic contents of STEM education. According to the keyword time zone map (Figure 6), STEM Education started to appear in 2004, which may be due to the fact that articles from the earlier literature were not included in the SCI-E and SSCI databases. Among the top 20 high-frequency keywords, after excluding the search term STEM Education, the four high-frequency keywords of student, gender, science, and education have relatively high betweenness centrality, and they play the role of “bridge” in STEM education research. In addition, the 28th ranked keyword “engineering education” has the highest betweenness centrality score with the high-frequency keyword “student,” which reflects the importance of engineering education in STEM education research. In each topic area, the articles were ranked from highest to lowest citation frequency, and the representative articles that were more closely related to the topic were selected for analysis of their research content (Wu et al., 2019).

**Table 3:** Top 20 high-frequency keywords.

Number	Count	Centrality	Keywords	Number	Count	Centrality	Keywords
1	387	0.11	Stem education	11	60	0.06	Science education
2	273	0.08	Science	12	56	0.02	Model
3	134	0.08	Education	13	52	0.07	Women
4	131	0.12	Student	14	49	0.03	Teacher
5	95	0.03	Mathematics	15	48	0.05	Impact
6	90	0.06	Performance	16	47	0.07	Design
7	71	0.04	Achievement	17	47	0.03	Professional development
8	71	0.06	Knowledge	18	45	0.06	School
9	65	0.02	Technology	19	44	0.04	Attitude
10	62	0.09	Gender	20	42	0.01	Motivation

Based on the number of occurrences and betweenness centrality scores of the top 20 high-frequency keywords, it can be seen that science, engineering education, student, and gender are the research hotspots in STEM education. These 20 high-frequency keywords were divided into four categories: STEM education components, STEM education teacher professional development, STEM education gender differences, and the impact of STEM education on students’ affective experiences (attitudes, self-efficacy, learning motivation, etc). Among the STEM education studies, there are more studies about the four areas of Science, Mathematics, Technology and Engineering Education. A complete engineering design is an emergent, highly iterative process that promotes meaningful learning (Roehrig et al., 2012; English, 2016b). It provides a framework that allows for a closer connection between the various disciplines of STEM education (Fan and Yu, 2017). Shahali et al. (2016) proposed that engineering design includes processes such as questioning, imagining, creating, testing, and improving, but is not limited to these processes. The implementation of engineering design requires scientific and mathematical concepts, so it can be used as a basis for establishing the connection between concept and practice in STEM education (Sanders, 2008; Donna, 2012). Among the studies on STEM education, there are more studies about students’ emotional experiences. According to Hwang et al. (2016), AR-based game teaching methods can improve students’ learning attitudes and learning outcomes. Su and Cheng (2013) found through the implementation of their experiment that the 3D GBL systems and software engineering course resulted in better student performance and motivation compared to traditional instruction. In addition, the professional development of STEM education teachers and gender differences in STEM education have been hot topics of interest to scholars.

The keyword time zone mapping (Figure 6) can reflect the research hotspots and their evolution trends in the STEM research field between 2004 and April 17, 2022. Since 2004, the evolution of research hotspots in STEM has been divided into three main phases: From 2004-to 2009, the field of STEM education focused on its component parts, with research on science and mathematics emerging one after another. From 2009-to 2015, the field of STEM education focused on student performance, self-efficacy, motivation, and gender differences (Wang and Degol, 2013; Freeman et al., 2014). During the period from 2015 to the present (April 17, 2022), research in the STEM field has focused on teacher professional development and the implementation of STEM education (Chai, 2019). We can predict that the hot topics of STEM education research in the coming year are still closely related to gender discrimination, teacher professional development, and sustainability of STEM education development.

### 3.3. Analysis of the research frontiers of science, technology, engineering, and mathematics education

**Figure 7:**
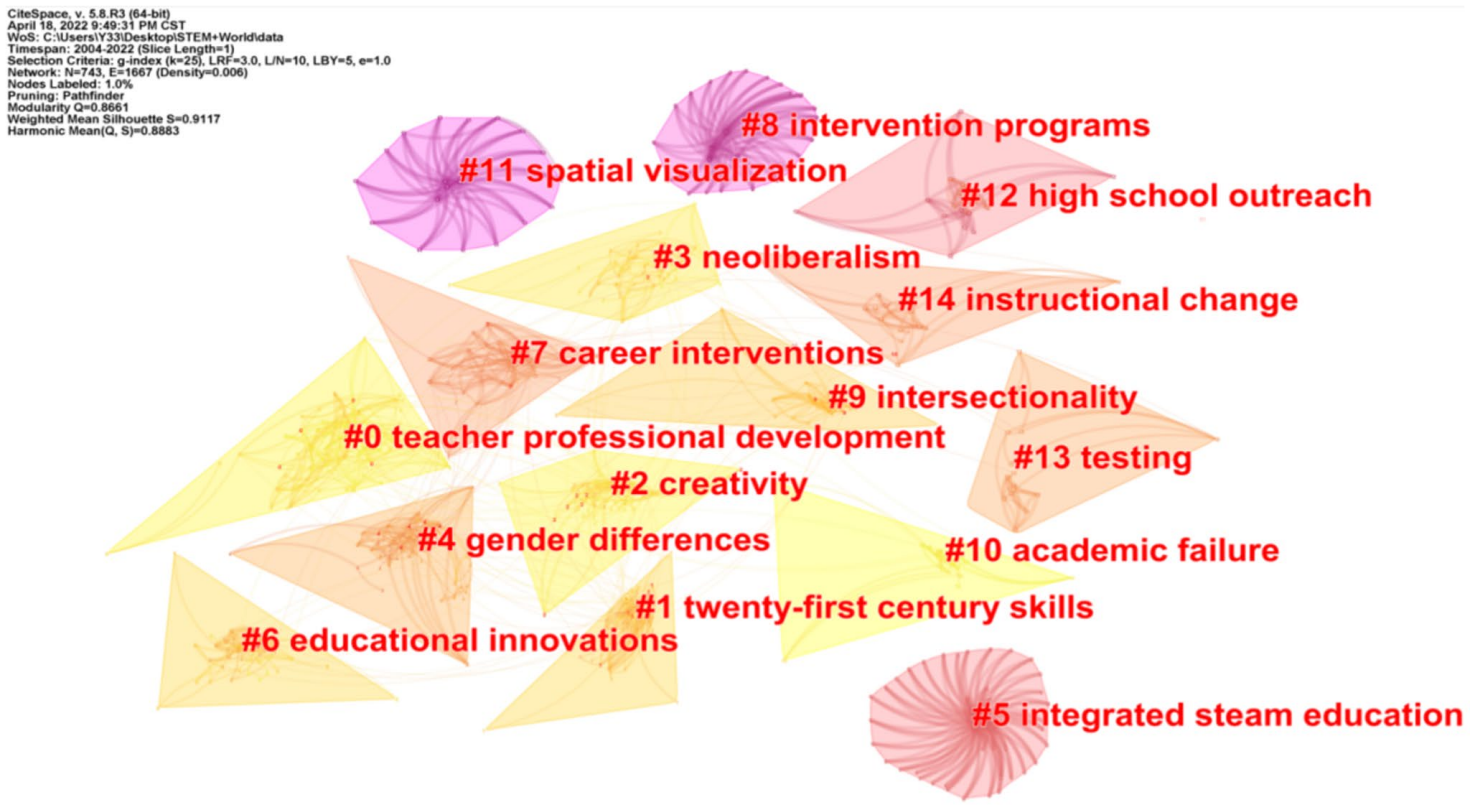
Clustering of co-cited references.

The co-citation clustering view of STEM education is generated using the cited references as nodes (Klavans and Boyack, 2017). In Figure 7, the Q value is 0.8661 and the S value is 0.9117, reflecting a good clustering effect. To maintain the clarity of the clusters, only the 15 clusters with a high number of citations and high homogeneity are shown in the figure, then 4 clusters are selected from them, and the highly correlated terms derived from these 4 clusters are summarized (Table 4). References frequently cited in the clusters were carefully studied to extract information on the frontiers of research. Then, the research frontiers of STEM education were derived by interpreting the research on the foundational topics of STEM education during the period 2004 to April 17, 2022, based on the time zone map of keyword co-occurrence. We summarize and extract them into the following four areas.

**Table 4:** High-frequency co-words for co-citation clustering.

Cluster ID	Size	Silhouette	Mean	Core noun terms
0	67	0.881	2018	Teacher professional development; science teachers; teachers; attitudes; and teacher leadership
1	63	0.906	2013	Twenty-first century skills; mathematical modelling; numeracy; k-12 education; and stem integration
2	55	0.924	2016	Creativity; integrated steam education; early childhood education; nature of stem; and steam education
4	48	0.971	2011	Gender differences; stem reform; stem persistence; transcript analysis; and upward transfer

#### 3.3.1. Research on the professional development of science, technology, engineering, and mathematics teachers

STEM, as a diversified educational model, plays an important role in the professional development of teachers. In the report of the *2030 Agenda for Sustainable Development*, teachers are emphasized as the cornerstone of improving education (Khuyen et al., 2020). Teachers are a key factor in determining the success or failure of STEM education implementation (Wang et al., 2011). Therefore, with the development of STEM education, the professional development of STEM education teachers has been of great interest to researchers. Researchers have argued that STEM education teaching practices are strongly influenced by STEM teachers’ perceptions (Wang et al., 2011; Park et al., 2016; Thibaut et al., 2018). Teachers with positive attitudes toward STEM education can drive STEM education implementation. Park et al. (2016) noted that experienced teachers have more positive attitudes than novice teachers in STEM education, but mathematics teachers are under-motivated in STEM teaching (Thibaut et al., 2018). Mathematics teachers find it more challenging to help students solve authentic problems in STEM instruction compared to science teachers (Wang et al., 2011). STEM teaching can be challenging for teachers, leading some colleges and universities to set out to develop professionals adapted to STEM education. Under the influence of the STEM education model, teachers are gradually moving toward interdisciplinary and diversified paths in their professional development.

#### 3.3.2. Research on science, technology, engineering, and mathematics education to develop students’ 21st-century skills

With the rise of STEM education, many researchers have also started to focus on how to develop 21st-century skills in STEM education. STEM education, as a curricular movement in American schools, is particularly focused on developing students’ 21st-century skills (Oretta, 2012). Common to STEM education and 21st-century skills is the ability to think critically, analyze problems, and solve them (Rifandi and Rahmi, 2019), each of which can be enhanced by using mathematics as a “booster” (Bergsten and Frejd, 2019). In the discussion on how mathematics education should prepare students for the future society, Gravemeijer et al. (2017) argued that 21st-century skills should be the goal of mathematics education (Capraro et al., 2013). Kertil and Gurel (2016) pointed out that mathematical modeling is a bridge to STEM education (Sayary and Adel, 2014). English (2016a) pointed out that modeling is a powerful tool for introducing features of 21st-century problems into the mathematics classroom. Mathematical modeling also developed students’ 21st-century skills (English, 2016b; Ärlebäck and Doerr, 2018). In addition, some researchers believed that the goal of STEM education is to develop 21st-century skills and that project-based learning approaches were more likely to develop 21st-century skills in the process of STEM education (Gravemeijer et al., 2017; Maass et al., 2019). While Sayary and Adel (2014) argued that problem-based learning strategies were more effective than project-based learning strategies and that problem-based learning approaches were the best way to enhance students’ 21st-century skills in STEM education (Kertil and Gurel, 2016).

#### 3.3.3. Research on science, technology, engineering, and mathematics education to foster creativity in early childhood

Creativity is one of the components of 21st-century skills and is emphasized in science education curricula in Turkey as well as in other countries (Altan and Tan, 2021). In terms of creativity, early childhood is a prime time to develop one’s creativity (Alfonso-Benlliure et al., 2013). Exposing children to STEM education at an early age can stimulate their curiosity and interest in STEM careers and foster their creativity (Kim et al., 2014). Alfuhaigi (2014) stated that a rich school environment has a positive impact on children’s developing creativity. Based on the fact that STEM education provides a rich educational environment for children (Corlu et al., 2014). Üret and Ceylan (2021) concluded that the impact of STEM education on the creativity of 5-year-old kindergarteners was positive and that this impact was permanent in exploring the impact of STEM education on the creativity of 5-year-old children attending kindergarten. Other researchers in similar studies have also concluded that STEM education has a positive effect on developing students’ creativity (Mayasari et al., 2016; Aguilera and Ortiz-Revilla, 2021). In addition, Shen et al. (2021) suggested that teachers providing feedback suggestions to students during STEM education is one of the key conditions for developing students’ creativity. Keana and Keana (2016) suggest design-based learning in STEM education as a way to develop creativity. Siew (2017) pointed out that the design process approach of STEM-Engineering can be used as a means to develop creativity, problem-solving skills, and thinking skills in rural secondary school students. Other researchers have suggested that the use of arts and crafts as an adjunct to STEM education can foster creativity in talented students (Root-Bernstein, 2015). STEM education takes a systematic approach to problem-solving with the help of science, technology, engineering, mathematics, and creativity (Stone-MacDonald et al., 2015). Thus, creativity and STEM education influence each other. Creativity participates in STEM education, and STEM education promotes the development of students’ creativity.

#### 3.3.4. Research on gender differences in science, technology, engineering, and mathematics education

Gender differences are one of the hot spots and focuses of research in the field of STEM education. Over the past 40 years, many countries have made significant progress and improvements in gender equality in science. A growing number of women are now earning STEM credentials and pursuing STEM careers, making outstanding contributions to enriching STEM knowledge and research (Dasgupta and Stout, 2014). However, while the gender gap in education has narrowed, there are still significant gender disparities in STEM education. Factors that may contribute to the problem are the environment, the school, and the students (Eddy and Brownell, 2016). Most students develop an interest in STEM careers during their secondary school years, with teachers and parents being the two most influential roles for students at an early age (Tey et al., 2020). However, there are differences in teachers’ attitudes toward male and female students in the STEM education process. Although teachers’ gender bias manifests itself more subtly when dealing with students, girls’ long-term exposure to the implication of teachers’ gender differences inevitably affects their self-confidence and has a negative impact on their development (Lindberg et al., 2010). Reinking and Martin (2018) argue that even though the number of women currently earning bachelor’s, master’s, or doctoral degrees in STEM fields in the United States is increasing each year, and women are performing as well or better than their male counterparts in STEM-related fields, women are losing interest in STEM and experiencing “leaky pipeline,” a steady loss of women from academic careers because men have better employment opportunities than women (Lindberg et al., 2010). Drury et al. (2011) argued that setting female role models in STEM is one of the most effective ways to prevent women from losing interest in STEM subjects. Much effort is needed to achieve gender equality and change the status of women in STEM fields, of which the provision of equitable resources and opportunities is crucial (van den Hurk et al., 2019). Improving a country’s competitiveness and achieving sustainable development requires harnessing the capabilities of all people, regardless of their gender.

### 3.4. Trends in science, technology, engineering, and mathematics education research topics in the last 18 years

**Figure 8:**
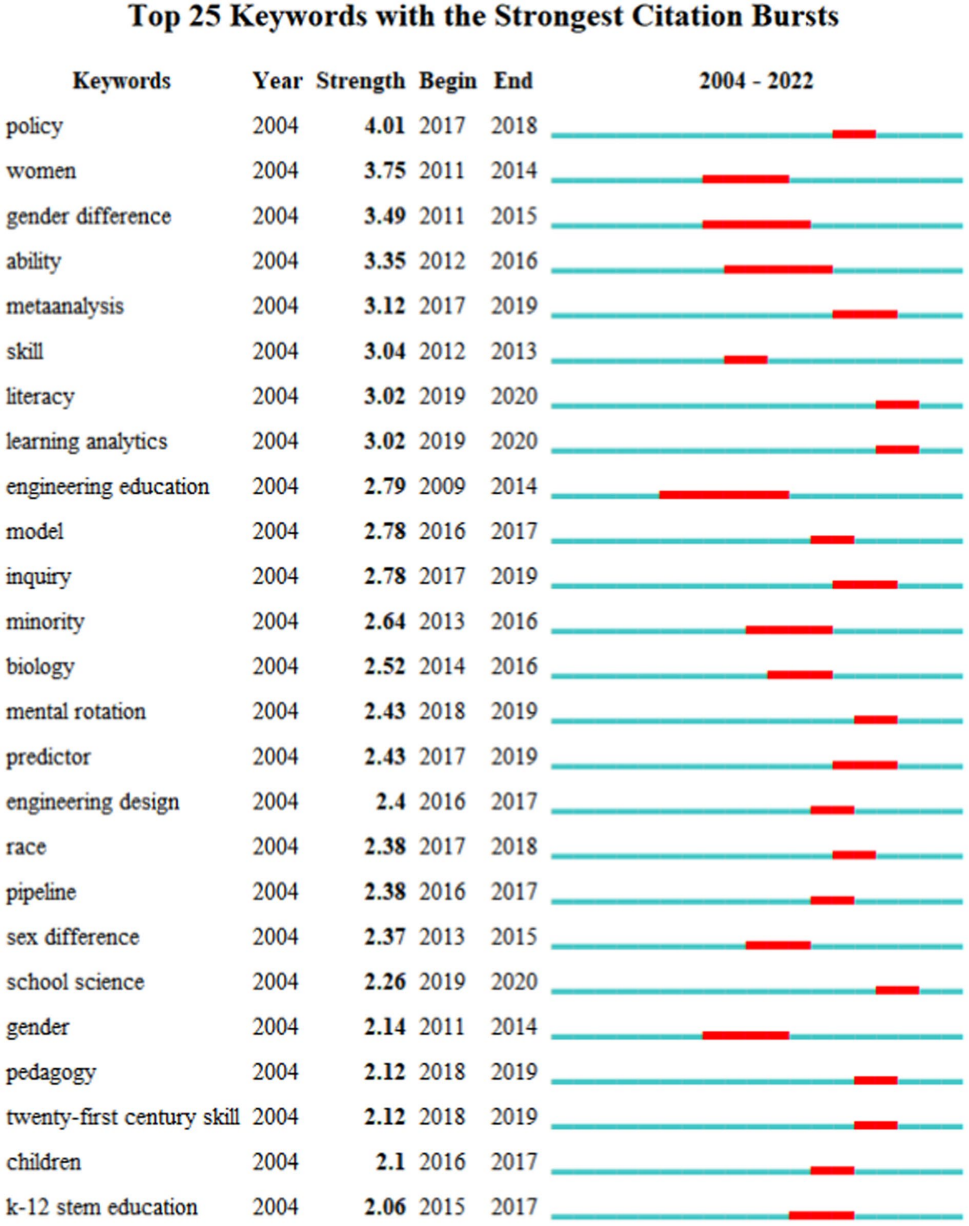
Mutability analysis of the first 25 high-frequency keywords.

Trends in STEM education research were analyzed by the burst keyword analysis method [indicating the tendency of keywords to change rapidly or increase sharply in number within a certain period, emphasizing the intensity of sudden keyword changes (Wei et al., 2020)]. The analysis of keywords that suddenly increase at a certain time point allows for the general identification of research trends on the timeline (Ordun et al., 2020). The top 25 burst keywords in STEM education research are listed in Figure 8. It can be seen that they all have an intensity of 2.0 or higher, with the highest reaching 4.01.

From 2004 to April 17, 2022, STEM education has been particularly popular in educational research. In terms of research content, studies prior to 2014 focused on gender differences and engineering education. Talent skill development in the 21st century and related issues have become one of the main areas of STEM education research, especially STEM education research in economically well-off regions and the development of STEM education in general cities has a greater impact on the development of students’ STEM literacy (Xie et al., 2015). STEM education influenced by big data and information technology is a trending research topic in recent years. With the increased demands of key technologies in STEM education, students’ problem-solving skills, creativity, and teachers’ professional development, literacy enhancement, and other literacies related to STEM education have become a new research focus (Chen et al., 2012).

From 2004 to 2016, gender difference and the demand for social jobs have attracted lots of attention from scholars on keywords such as “gender difference,” “skill” and “ability.” From 2016 to 2018, the strongest mutation is “policy,” which corresponds to the development path of STEM education, i.e., the development of STEM education is facilitated by the policies issued by governments about STEM education. After 2018, STEM education has ushered in new opportunities with the continuous development and improvement of big data technology and artificial intelligence technology. During this period, the strongest mutations were in “learning analytics” and “literacy.” Scholars have explored artificial intelligence-based learning analytics and STEM literacy as key research components in STEM education.

## 4. Discussion

In this paper, we use CiteSpace (5.8.R3) to visually analyze 1,420 papers related to STEM education, which helps us to systematically understand the status of the STEM education research field. This method can, to a certain extent, better overcome the limitations of literature analysis and reduce the subjectivity and difficulty of manual screening. In this study, we perform a visual analysis of the number of publications, authors, countries, research institutions, and keywords. The results showed that there has been a significant increase since 2010 in terms of the number of publications, and this result is similar to other STEM education trend studies (Ha et al., 2020; Li et al., 2020; Takeuchi et al., 2020). For example, Gil-Doménech et al. (2018) analyzed 747 papers obtained from the Web of Science database between 1991 and 2016. Their study showed that most papers on STEM education were published after 2010 and that the number of publications has increased significantly in recent years. This may be due to the growing interest in STEM education among researchers worldwide (Gil-Doménech et al., 2018; Li et al., 2019).

In terms of the distribution of countries and research institutions, the United States was the country with the highest number of publications between 2004 and April 17, 2022, among the 77 countries involved in the literature, and the United States accounted for the majority of institutions concerned with STEM education research. This is consistent with the findings of other studies (Gil-Doménech et al., 2018; Li et al., 2019; Özkaya, 2019). Özkaya (2019) analyzed 2,313 papers extracted from the Web of Science database between 1992 and 2017 and concluded that the United States contributed the most to STEM education research, followed by the United Kingdom and Australia. Gil-Doménech et al. (2018) studied that the 15 institutions with a high number of publications in STEM education all originated from the United States These results are inextricably linked to a large number of state and government policies enacted to support the development of STEM education in the United States since its inception. However, compared to the United States, China has only one-ninth the number of publications and few research institutions. This is also related to the lack of national top-level design; therefore, China needs to learn from international STEM education development strategies to promote the development of STEM education in China. In addition, STEM education research is fragmented. A reading and analysis of the literature reveal that most domestic and international research on STEM education has been conducted in colleges and universities, with teachers and students as groups forming relatively independent research groups and a lack of cooperation among research institutions, experts, and scholars (Min et al., 2020).

Based on the high-frequency keywords, it is seen that the research hotspots in STEM education mainly focus on the professional development of STEM education teachers, gender differences in STEM education, and the effects of STEM education on students’ affective experiences (attitudes, self-efficacy, and learning motivation, etc). A strong faculty is a guarantee for the successful implementation of STEM education; therefore, the professional development of STEM education teachers has received extensive attention from researchers and has become a hot spot in the field of STEM research (Chai, 2019; Chiu et al., 2021; Lo, 2021). The goal of STEM education is to develop 21st-century human resources with creativity, critical thinking, and problem-solving skills. For STEM education to be implemented more effectively, good emotional experiences for students during STEM teaching and learning are crucial. STEM education has a significant impact on students’ attitudes and learning outcomes compared to traditional courses (Huang, 2020). In addition, if students’ interest in STEM careers is fostered from childhood, it facilitates students’ later development in STEM courses and plays an important role in the emotional experience of female students especially in terms of self-efficacy and motivation to learn (Tzu-Ling, 2019; Kryshko et al., 2022).

Although this study systematically analyzed the latest developments in STEM education research, it still had some limitations. For example, the number of documents analyzed was limited. We only analyzed articles in SSCI and SCI-E databases and all of them were written in English, which neglected articles written in other languages and included in other databases, and the depth and comprehensiveness of the analysis were not sufficient. In addition, we only analyzed the literature and lacked some empirical studies of the literature findings. When applying the CiteSpace tool for co-citation clustering, there were 15 cluster samples, but we only analyzed the study paths for four clusters with high citation counts and high homogeneity, and the analysis was somewhat subjective. Understanding more and more specific research paths require more intensive reading of the literature and more in-depth research and analysis based on this foundation.

## 5. Conclusion

STEM education has received extensive attention from international scholars in the field of education. In this study, it is concluded that in terms of the distribution of countries and research institutions, the United States is the country with the largest number of publications, and the United States accounts for the majority of institutions related to STEM education research. The research hotspots of STEM education mainly focused on engineering education, teacher professional development, gender differences, etc. Research on the professional development of STEM teachers, the cultivation of 21st-century skills in STEM education, the cultivation of early childhood creativity in STEM education, and the gender differences in STEM education are the research frontiers and trends in recent years. After 2018, with the continuous development of big data technology and artificial intelligence technology, learning analysis and STEM literacy have also become the main research contents of STEM education in recent years. This research systematically analyzes the latest development of global STEM education research, which is beneficial for readers to understand the whole picture of STEM education research, and provides references for researchers to conduct further and targeted research.

## Data availability statement

The original contributions presented in the study are included in the article/supplementary material, further inquiries can be directed to the corresponding author.

## Author contributions

DY performed the data collection and analysis and completed the article. XW proposed the structure of the study. JL took charge of the language work and proofread the article. JZ gave some good advice. All authors have read and agreed to the published version of the manuscript.

## Funding

This work was supported by the National Natural Science Foundation of China (No. 61862051), Science and Technology Foundation of Guizhou Province (No. [2019]1299), Top-Notch Talent Program of Guizhou Province (No. KY[2018]080), Guizhou Educational Science Planning Project under Grant (No. 2021B201), Educational Department of Guizhou under Grant No. KY[2019]067, Qianan Educational Science Planning Project under Grant (No. 2021B001), Funds of Qiannan Normal University for Nationalities (No. 2021gh19), and Key Project of Jilin Provincial Education Planning: Opportunities and Challenges of Modernizing Basic Education in Jilin under the Process of Chinese Education Modernization.

## Conflict of interest

The authors declare that the research was conducted in the absence of any commercial or financial relationships that could be construed as a potential conflict of interest.

## Publisher’s note

All claims expressed in this article are solely those of the authors and do not necessarily represent those of their affiliated organizations, or those of the publisher, the editors and the reviewers. Any product that may be evaluated in this article, or claim that may be made by its manufacturer, is not guaranteed or endorsed by the publisher.

## References

[ref1] Aguilera D. Ortiz-Revilla J. (2021). STEM vs. STEAM education and student creativity: a systematic literature review. Educ. Sci. 11:331. doi: 10.3390/educsci11070331

[ref2] Aksela M. K. (2015). “Luma Centre Finland: the Finnish model to educate teachers in math, science and technology” in 10 Jahre LeLa Jahrestagung. eds. Heinichen U. Haupt O. J. (LernortLabor: Bundesverband der Schülerlabore), 16–18.

[ref3] Alfonso-Benlliure V. Meléndez J. C. García-Ballesteros M. (2013). Evaluation of a creativity intervention program for preschoolers. Think. Skills Creat. 10, 112–120. doi: 10.1016/j.tsc.2013.07.005

[ref4] Alfuhaigi S. (2014). “School Environment and Creativity Development: A Review of Literature,” in Proceedings of SITE 2014--Society for Information Technology & Teacher Education International Conference. eds. Searson M. Ochoa M. (Jacksonville, Florida, United States: Association for the Advancement of Computing in Education), 1832–1837.

[ref5] Allwood J. M. Skelton A. (2017). Industrial Metamorphosis: A Response to the Green Paper on Building Our Industrial Strategy. Cambridge, UK: Department of Engineering, University of Cambridge.

[ref6] Altan E. B. Tan S. (2021). Concepts of creativity in design-based learning in STEM education. Int. J. Technol. Des. Educ. 31, 503–529. doi: 10.1007/s10798-020-09569-y

[ref7] Ärlebäck J. B. Doerr H. M. (2018). Students’ interpretations and reasoning about phenomena with negative rates of change throughout a model development sequence. ZDM 50, 187–200. doi: 10.1007/s11858-017-0881-5

[ref8] Aslam F. Adefila A. Bagiya Y. (2018). STEM outreach activities: an approach to teachers’ professional development. J. Educ. Teach. 44, 58–70. doi: 10.1080/02607476.2018.1422618

[ref9] Augustine N. R. (2007). Is America Falling Off the Flat Earth? Washington, DC: National Academies Press.

[ref10] Barrett B. S. Moran A. L. Woods J. E. (2014). Meteorology meets engineering: an interdisciplinary STEM module for middle and early secondary school students. Int. J. STEM Educ. 1, 1–7. doi: 10.1186/2196-7822-1-6

[ref11] Bergsten C. Frejd P. (2019). Preparing pre-service mathematics teachers for STEM education: an analysis of lesson proposals. ZDM 51, 941–953. doi: 10.1007/s11858-019-01071-7

[ref12] Blättel-Mink B. (2009). MINT: the German national initiative for more women in SET: interview with Barbara Schwarze, coordinator of MINT, competence center technology-diversity-equal chances, Inc., Bielefeld, Germany. Equal. Oppor. Int. 28, 104–109. doi: 10.1108/02610150910933677

[ref13] Bo Z. (2022). International STEM education research Progress and enlightenment–based on the content analysis of the papers published in the SSCI journal international journal of STEM education. Journal of. Math. Educ. 31, 58–62. doi: 10.CNKI:SUN:SXYB.0.2022-02-010, (In Chinese)

[ref14] Capraro R. M. Capraro M. M. Morgan J. R. (2013). STEM Project-Based Learning: An Integrated Science, Technology, Engineering, and Mathematics (STEM) Approach, Vol. 2. The Netherlands: Sense Publishers.

[ref15] Chai C. S. (2019). Teacher professional development for science, technology, engineering and mathematics (STEM) education: a review from the perspectives of technological pedagogical content (TPACK). Asia Pac. Educ. Res. 28, 5–13. doi: 10.1007/s40299-018-0400-7

[ref16] Chen C. (2006). CiteSpace II: detecting and visualizing emerging trends and transient patterns in scientific literature. J. Am. Soc. Inf. Sci. Technol. 57, 359–377. doi: 10.1002/asi.20317

[ref17] Chen H. Chiang R. H. Storey V. C. (2012). Business intelligence and analytics: from big data to big impact. MIS Q. 36, 1165–1188. doi: 10.2307/41703503

[ref18] Chen C. Hu Z. Liu S. Tseng H. (2012). Emerging trends in regenerative medicine: a scientomanalysis in CiteSpace. Expert. Opin. Biol. Ther. 12, 593–608. doi: 10.1517/14712598.2012.674507, PMID: 22443895

[ref19] Chiu T. K. Chai C. S. Williams P. J. Lin T. J. (2021). Teacher professional development on self-determination theory–based design thinking in STEM education. Educational. Technol. Soc. 24, 153–165.

[ref20] Corbett D. (2014). Where is Australia's national STEM strategy? Eng. Australia 86, 42–51. doi: 10.3316/informit.981752640784091

[ref21] Corlu M. S. Capraro R. M. Capraro M. M. (2014). Introducing STEM education: implications for educating our teachers in the age of innovation. Eğitim ve Bilim 39, 74–85.

[ref22] Danni C. Qi Z. Jiahui Q. Liang C. Zhonglu G. (2018). Literature review on the role of root in soil erosion control based on the knowledge map. Sci. Soil Water Conserv. 16, 124–135. doi: 10.16843/j.sswc.2018.06.016, In Chinese

[ref23] Dasgupta N. Stout J. G. (2014). Girls and women in science, technology, engineering, and mathematics: STEMing the tide and broadening participation in STEM careers. Policy Insights Behav. Brain Sci. 1, 21–29. doi: 10.1177/2372732214549471

[ref24] Donna J. D. (2012). A model for professional development to promote engineering design as an integrative pedagogy within STEM education. J-PEER 2, 1–8. doi: 10.5703/1288284314866

[ref25] Drury B. J. Siy J. O. Cheryan S. (2011). When do female role models benefit women? The importance of differentiating recruitment from retention in STEM. Psychol. Inq. 22, 265–269. doi: 10.1080/1047840X.2011.620935

[ref26] Eddy S. L. Brownell S. E. (2016). Beneath the numbers: a review of gender disparities in undergraduate education across science, technology, engineering, and math disciplines. Phys. Rev. Phys. Educ. Res. 12:020106. doi: 10.1103/PhysRevPhysEducRes.12.020106

[ref27] English L. D. (2016a). “Advancing mathematics education research within a STEM environment,” in Research in Mathematics Education in Australasia 2012–2015. eds. Makar K. Dole S. Visnovska J. Goos M. Bennison A. Fry K. (Singapore: Springer), 353–371.

[ref28] English L. D. (2016b). STEM education K-12: perspectives on integration. Int. J. STEM Educ. 3, 1–8. doi: 10.1186/s40594-016-0036-1

[ref29] Fan S. C. Yu K. C. (2017). How an integrative STEM curriculum can benefit students in engineering design practices. Int. J. Technol. Des. Educ. 27, 107–129. doi: 10.1007/s10798-015-9328-x

[ref30] Freeman S. Eddy S. L. McDonough M. Smith M. K. Okoroafor N. Jordt H.. (2014). Active learning increases student performance in science, engineering, and mathematics. Proc. Natl. Acad. Sci. 111, 8410–8415. doi: 10.1073/pnas.1319030111, PMID: 24821756 PMC4060654

[ref31] Gazni A. Sugimoto C. R. Didegah F. (2012). Mapping world scientific collaboration: authors, institutions, and countries. J. Am. Soc. Inf. Sci. Technol. 63, 323–335. doi: 10.1002/asi.21688

[ref32] Gil-Doménech D. Berbegal-Mirabent J. Merigó J. M. (2018). “STEM education: a bibliometric overview” in International Conference on Modelling and Simulation in Management Sciences. eds. Ferrer-Comalat J. Linares-Mustarós S. Merigó J. Kacprzyk J. (Cham: Springer), 193–205.

[ref33] Gonzalez H. B. (2015). The America COMPETES acts: An overview. Library of Congress Congressional Research Service.

[ref34] Gravemeijer K. Stephan M. Julie C. Lin F. L. Ohtani M. (2017). What mathematics education may prepare students for the society of the future? Int. J. Sci. Math. Educ. 15, 105–123. doi: 10.1007/s10763-017-9814-6

[ref35] Ha C. T. Thao T. T. P. Trung N. T. Van Dinh N. Trung T. (2020). A bibliometric review of research on STEM education in ASEAN: science mapping the literature in Scopus database, 2000–2019. EURASIA J. Math. Sci. Technol. Educ. 16:em1889. doi: 10.29333/ejmste/8500

[ref36] Huang F. (2020). Effects of the application of STEAM education on students’ learning attitude and outcome- the case of Fujian Chuanzheng communications college. Revista de Cercetare şi Intervenţie Socială 69, 349–356. doi: 10.33788/rcis.69.23

[ref37] Hwang G. J. Wu P. H. Chen C. C. Tu N. T. (2016). Effects of an augmented reality-based educational game on students' learning achievements and attitudes in real-world observations. Interact. Learn. Environ. 24, 1895–1906. doi: 10.1080/10494820.2015.1057747

[ref38] Irwanto I. Saputro A. D. Ramadhan M. F. Lukman I. R. (2022). Research trends in STEM education from 2011 to 2020: a systematic review of publications in selected journals. Int. J. Int. Mob. Technol. 16, 19–32. doi: 10.3991/ijim.v16i05.27003

[ref39] Keana L. Keana M. (2016). STEAM by design. Design Technol. Educ. Int. J. 21, 61–82.

[ref40] Kennedy S. Johnson C. K. (2016). Perfecting China, Inc.: China's 13th Five-Year Plan. Washington, DC: Rowman & Littlefield.

[ref41] Kertil M. Gurel C. (2016). Mathematical modeling: a bridge to STEM education. Int. J. Educ. Math. Sci. Technol. 4, 44–55. doi: 10.18404/ijemst.95761

[ref42] Khuyen N. Bien N. V. Lin P. L. Lin J. Chang C. Y. (2020). Measuring teachers’ perceptions to sustain STEM education development. Sustainability 12:1531. doi: 10.3390/su12041531

[ref43] Kim D. H. Ko D. G. Han M. J. Hong S. H. (2014). The effects of science lessons applying STEAM education program on the creativity and interest levels of elementary students. J. Korean Assoc. Sci. Educ. 34, 43–54. doi: 10.14697/jkase.2014.34.1.1.00043

[ref44] Klavans R. Boyack K. W. (2017). Which type of citation analysis generates the most accurate taxonomy of scientific and technical knowledge? J. Assoc. Inf. Sci. Technol. 68, 984–998. doi: 10.1002/asi.23734

[ref45] Kryshko O. Fleischer J. Grunschel C. Leutner D. (2022). Self-efficacy for motivational regulation and satisfaction with academic studies in STEM undergraduates: the mediating role of study motivation. Learn. Individ. Differ. 93:102096. doi: 10.1016/j.lindif.2021.102096

[ref46] Li H. An H. Wang Y. Huang J. Gao X. (2016). Evolutionary features of academic articles co-keyword network and keywords co-occurrence network: based on two-mode affiliation network. Physica A 450, 657–669. doi: 10.1016/j.physa.2016.01.017

[ref47] Li Y. Froyd J. E. Wang K. (2019). Learning about research and readership development in STEM education: a systematic analysis of the journal’s publications from 2014 to 2018. Int. J. STEM Educ. 6, 1–8. doi: 10.1186/s40594-019-0176-1

[ref48] Li Y. Schoenfeld A. H. (2019). Problematizing teaching and learning mathematics as “given” in STEM education. Int. J. STEM Educ. 6, 1–13. doi: 10.1186/s40594-019-0197-9

[ref49] Li Y. Wang K. Xiao Y. Froyd J. E. (2020). Research and trends in STEM education: a systematic review of journal publications. Int. J. STEM Educ. 7, 1–16. doi: 10.1186/s40594-020-00207-6

[ref50] Lindberg S. M. Hyde J. S. Petersen J. L. Linn M. C. (2010). New trends in gender and mathematics performance: a meta-analysis. Psychol. Bull. 136, 1123–1135. doi: 10.1037/a0021276, PMID: 21038941 PMC3057475

[ref51] Liu X. Liu N. Zhou M. Lu Y. Li F. (2018). Bibliometric analysis of global research on the rehabilitation of spinal cord injury in the past two decades. Ther. Clin. Risk Manag. 15, 1–14. doi: 10.2147/TCRM.S163881, PMID: 30588000 PMC6301731

[ref52] Lo C. K. (2021). Design principles for effective teacher professional development in integrated STEM education: a systematic review. Educ. Technol. Soc. 24, 136–152.

[ref53] Maass K. Geiger V. Ariza M. R. Goos M. (2019). The role of mathematics in interdisciplinary STEM education. ZDM 51, 869–884. doi: 10.1007/s11858-019-01100-5

[ref54] Marginson S. Tytler R. Freeman B. Roberts K. (2013). STEM: Country Comparisons: International Comparisons of Science, Technology, Engineering and Mathematics (STEM) Education Final Report. Melbourne: Australian Council of Learned Academies.

[ref55] Mayasari T. Kadarohman A. Rusdiana D. Kaniawati I. (2016). “Exploration of student’s creativity by integrating STEM knowledge into creative products,” in AIP Conference Proceedings (United States: AIP Publishing LLC), 1708:080005.

[ref56] Min L. I. Jingpeng L. I. U. Yushun L. I. (2020). “A comparative analysis of STEM education research (2016–2020) based on CiteSpace,” in In 2020 Ninth International Conference of Educational Innovation Through Technology (EITT). (Porto, Portugal: IEEE), 128–132.

[ref57] Murphy S. MacDonald A. Danaia L. Wang C. (2019). An analysis of Australian STEM education strategies. Policy Fut. Educ. 17, 122–139. doi: 10.1177/1478210318774190

[ref58] Ordun C. Purushotham S. Raff E. (2020). Exploratory Analysis of Covid-19 Tweets using Topic Modeling, Umap, and Digraphs. Los Alamos, United States: arXiv.

[ref59] Oretta C. (2012). 21st Century Skills Practices and Programs: A Case Study at An Elementary School. ProQuest LLC: Ann Arbor, MI: University of Southern California ProQuest Dissertations Publishing.

[ref60] Özkaya A. (2019). Bibliometric analysis of the publications made in STEM education area. Bartin Üniversitesi Egitim Fakültesi Dergisi 8, 590–628. doi: 10.14686/buefad.450825

[ref61] Park H. Byun S. Y. Sim J. Han H. S. Baek Y. S. (2016). Teachers’ perceptions and practices of STEAM education in South Korea. Eurasia J. Math. Sci. Technol. Educ. 12, 1739–1753. doi: 10.12973/eurasia.2016.1531a

[ref62] Parsons W. (2002). From muddling through to muddling up-evidence based policy making and the modernization of British government. Public Policy Admin. 17, 43–60. doi: 10.1177/095207670201700304

[ref63] Reinking A. Martin B. (2018). The gender gap in STEM fields: theories, movements, and ideas to engage girls in STEM. J. New Appr. Educ. Res. 7, 148–153. doi: 10.7821/naer.2018.7.271

[ref64] Rifandi R. Rahmi Y. L. (2019). “STEM education to fulfil the 21st century demand: a literature review,” in Journal of Physics: Conference Series (England and Wales: IOP Publishing), 1317:012208.

[ref65] Roehrig G. H. Moore T. J. Wang H. H. Park M. S. (2012). Is adding the E enough? Investigating the impact of K-12 engineering standards on the implementation of STEM integration. Sch. Sci. Math. 112, 31–44. doi: 10.1111/j.1949-8594.2011.00112.x

[ref66] Root-Bernstein R. (2015). Arts and crafts as adjuncts to STEM education to foster creativity in gifted and talented students. Asia Pac. Educ. Rev. 16, 203–212. doi: 10.1007/s12564-015-9362-0

[ref67] Ruifang X. (2017). Exploration and practice of STEM curricula in science and technology museums: taking the case of “STEM wonderful day in Shanghai Science & Technology Museum”. Sci. Educ. Mus. 3, 100–106. doi: 10.16703/j.cnki.31-2111/n.2017.02.004, In Chinese

[ref68] Sanders M. E. (2008). Stem, stem education, stemmania. Technol. Teach. 68, 20–26.

[ref69] Sayary E. Adel A. M. (2014). The effectiveness of problem-based learning strategy in STEM education for enhancing students’ 21st century-skills [doctoral dissertation, the British University in Dubai (BUiD)]. The United Arab Emirates: The British Universityn Dubai.

[ref70] Schneider J. W. (2004). Mapping scientific frontiers: the quest for knowledge visualization. J. Assoc. Inf. Sci. Technol. 55, 363–365. doi: 10.1002/asi.10383

[ref71] Shahali E. H. M. Halim L. Rasul M. S. Osman K. Zulkifeli M. A. (2016). STEM learning through engineering design: impact on middle secondary students’ interest towards STEM. EURASIA J. Math. Sci. Technol. Educ. 13, 1189–1211. doi: 10.12973/eurasia.2017.00667a

[ref72] Shapley D. (2000). The national science board: A history in highlights, 1950–2000. National Science Foundation, National Science Board, 4201 Wilson Blvd. Arlington, VA.

[ref73] Sharma J. Yarlagadda P. K. (2018). Perspectives of ‘STEM education and policies’ for the development of a skilled workforce in Australia and India. Int. J. Sci. Educ. 40, 1999–2022. doi: 10.1080/09500693.2018.1517239

[ref74] Shen S. Wang S. Qi Y. Wang Y. Yan X. (2021). Teacher suggestion feedback facilitates creativity of students in STEAM education. Front. Psychol. 12:723171. doi: 10.3389/fpsyg.2021.723171, PMID: 34539525 PMC8441010

[ref75] Siew N. M. (2017). Integrating STEM in an engineering design process: the learning experience of rural secondary school students in an outreach challenge program. Eurasia Proc. Educ. Soc. Sci. 6, 128–141.

[ref76] Smith E. (2010). Is there a crisis in school science education in the United Kingdom? Educ. Rev. 62, 189–202. doi: 10.1080/00131911003637014

[ref77] Stine D. D. (2009). America COMPETES Act: Programs, Funding, and Selected Issues. Washington DC: Library of Congress Washington DC Congressional Research Service.

[ref78] Stone-MacDonald A. Wendell K. Douglass A. Love M. L. (2015). Engaging Young Engineers: Teaching Problem Solving Skills Through STEM. Baltimore: Brookes Publishing.

[ref79] Su W. (2017). Interpretation of the 2017 white paper on STEM education in China. Mod. Educ. 19, 4–7. doi: 10.CNKI:SUN:XDJU.0.2017-07-003, In Chinese

[ref80] Su C. H. Cheng C. H. (2013). 3D game-based learning system for improving learning achievement in software engineering curriculum. TOJET 12, 1–12.

[ref81] Takeuchi M. A. Sengupta P. Shanahan M. C. Adams J. D. Hachem M. (2020). Transdisciplinarity in STEM education: a critical review. Stud. Sci. Educ. 56, 213–253. doi: 10.1080/03057267.2020.1755802

[ref82] Tang M. Liao H. Wan Z. Herrera-Viedma E. Rosen M. A. (2018). Ten years of sustainability (2009 to 2018): a bibliometric overview. Sustainability 10:1655. doi: 10.3390/su10051655

[ref83] Tey T. C. Y. Moses P. Cheah P. K. (2020). Teacher, parental and friend influences on STEM interest and career choice intention. Iss. Educ. Res. 30, 1558–1575. doi: 10.3316/informit.606484600109604

[ref84] Thibaut L. Knipprath H. Dehaene W. Depaepe F. (2018). How school context and personal factors relate to teachers’ attitudes toward teaching integrated STEM. Int. J. Technol. Des. Educ. 28, 631–651. doi: 10.1007/s10798-017-9416-1

[ref85] Tzu-Ling H. (2019). Gender differences in high-school learning experiences, motivation, self-efficacy, and career aspirations among Taiwanese STEM college students. Int. J. Sci. Educ. 41, 1870–1884. doi: 10.1080/09500693.2019.1645963

[ref86] Upham S. Small H. (2010). Emerging research fronts in science and technology: patterns of new knowledge development. Scientometrics 83, 15–38. doi: 10.1007/s11192-009-0051-9, PMID: 32214555 PMC7088980

[ref87] Üret A. Ceylan R. (2021). Exploring the effectiveness of STEM education on the creativity of 5-year-old kindergarten children. Eur. Early Child. Educ. Res. J. 29, 842–855. doi: 10.1080/1350293X.2021.1913204

[ref88] van den Hurk A. Meelissen M. van Langen A. (2019). Interventions in education to prevent STEM pipeline leakage. Int. J. Sci. Educ. 41, 150–164. doi: 10.1080/09500693.2018.1540897

[ref89] Vihma L. Aksela M. (2014). “Inspiration, joy, and support of STEM for children, youth, and teachers through the innovative LUMA collaboration” in Finnish innovations and technologies in schools (The Netherlands: Brill), 129–144.

[ref90] Wang M. T. Degol J. (2013). Motivational pathways to STEM career choices: using expectancy–value perspective to understand individual and gender differences in STEM fields. Dev. Rev. 33, 304–340. doi: 10.1016/j.dr.2013.08.001, PMID: 24298199 PMC3843492

[ref91] Wang H. H. Moore T. J. Roehrig G. H. Park M. S. (2011). STEM integration: teacher perceptions and practice. J-PEER 1, 1–13. doi: 10.5703/1288284314636

[ref92] Wei J. Liang G. Alex J. Zhang T. Ma C. (2020). Research progress of energy utilization of agricultural waste in China: Bibliometric analysis by CiteSpace. Sustainability 12:812. doi: 10.3390/su12030812

[ref93] Wu Y. Wang H. Wang Z. Zhang B. Meyer B. C. (2019). Knowledge mapping analysis of rural landscape using CiteSpace. Sustainability 12, 1–17. doi: 10.3390/su12010066

[ref94] Xie Y. Fang M. Shauman K. (2015). STEM education. Annu. Rev. Sociol. 41, 331–357. doi: 10.1146/annurev-soc-071312-145659, PMID: 26778893 PMC4712712

[ref95] Xiuling S. Xiuping T. Bo F. Fucheng M. (2017). A review of domestic research on STEM education. Sci. Pop. 81, 3–4. doi: 10.16728/j.cnki.kxdz.2017.07.001, In Chinese

[ref96] Ying Z. (2021). The current status of domestic STEAM education research: an analysis based on the social network mapping of CSSCI literature from 2000 to 2020. Primary Mid. School Educ. Technol. 44, 6–10. doi: 10.CNKI:SUN:ZXDJ.0.2021-11-003, In Chinese

[ref97] Zhang J. Cenci J. Becue V. Koutra S. Ioakimidis C. S. (2020). Recent evolution of research on industrial heritage in Western Europe and China based on bibliometric analysis. Sustainability 12, 1–15. doi: 10.3390/su1213534835136666

[ref98] Zhang Q. Rong G. Meng Q. Yu M. Xie Q. Fang J. (2020). Outlining the keyword co-occurrence trends in Shuanghuanglian injection research: a bibliometric study using CiteSpace III. J. Trad. Chin. Med. Sci. 7, 189–198. doi: 10.1016/j.jtcms.2020.05.006

